# Spatial Omics Sequencing Based on Microfluidic Array Chips

**DOI:** 10.3390/bios13070712

**Published:** 2023-07-06

**Authors:** Jianyu Shi, Yating Pan, Xudong Liu, Wenjian Cao, Ying Mu, Qiangyuan Zhu

**Affiliations:** State Key Laboratory of Industrial Control Technology, Research Center for Analytical Instrumentation, Institute of Cyber-Systems and Control, College of Control Science and Engineering, Zhejiang University, Hangzhou 310000, China; shijianyu2022@zju.edu.cn (J.S.); 22007053@zju.edu.cn (Y.P.); lxd112@zju.edu.cn (X.L.); wenjian.cao@zju.edu.cn (W.C.)

**Keywords:** spatial transcriptomics, spatial multi-omics sequencing, *in situ* capture and sequencing, single-cell sequencing, microfluidic array chip

## Abstract

Spatial profiling technologies fill the gap left by the loss of spatial information in traditional single-cell sequencing, showing great application prospects. After just a few years of quick development, spatial profiling technologies have made great progress in resolution and simplicity. This review introduces the development of spatial omics sequencing based on microfluidic array chips and describes barcoding strategies using various microfluidic designs with simplicity and efficiency. At the same time, the pros and cons of each strategy are compared. Moreover, commercialized solutions for spatial profiling are also introduced. In the end, the future perspective of spatial omics sequencing and research directions are discussed.

## 1. Introduction

Traditional single-cell sequencing technologies rely on tissue dissociation in the acquisition of single cells, which abandons the spatial positional information of different types of cells. However, the growth and differentiation of cells are influenced by the environment in which they are located. Therefore, ignoring the spatial location information cannot accurately determine the microenvironment of the cells, which is not conducive to the subsequent analysis of cell function. The development of spatial omics technology has filled in the blank areas of cellular organization and interactions in omics analysis. Meanwhile, it has unique advantages in the analysis of specific gene expression and cell localization and can provide key information for the research of detailed molecular mechanisms.

Therefore, positioning cells by spatial omics technology can explore the differentiation trajectory and development of cell lineages [1,2], which may have great application prospects in developmental biology. At the same time, spatial profiling technology can directly profile the heterogeneity of gene expression in different tissue regions, making it suitable for studying the heterogeneity of malignant tumors [3,4,5]. The utilization of spatial omics technology can associate transcriptomes with morphology and physiology when determining cell types, thus constructing a more precise spatial cell subtype map. Therefore, spatial omics technology has many contributions to make in the study of brain neural circuits [6,7,8]. Additionally, spatial sequencing technology also has new applications in the fields of novel coronavirus infection immune response [9], viral infection [10], plant tissue research [11], and so on. 

In the year 2019, Nature Reviews Genetics published an article entitled ‘Spatial transcriptomics coming of age’ [12], evaluating that spatial transcriptomics technology has great potential in providing molecular maps of various tissue systems, officially opening the prelude to spatial transcriptome sequencing. At the beginning of the year 2021, the journal Nature Methods named spatially resolved transcriptomics as the technology of the year 2020 [13], which shows the great potential and influence of spatial omics technology.

## 2. General Classification of Spatial Profiling Technology

In general, spatial resolution transcriptomics is mainly divided into two categories: One is an image-based method based on *in situ* hybridization, in which staining and barcoding RNA in its original position are the key points to achieve multiple detection. The second category is based on *in situ* capture and sequencing: the spatial information is marked by barcoded primers attached to a solid surface, and then the spatial information is retrieved by high-throughput sequencing. Image-based methods originating from single-molecule RNA *in situ* hybridization are mainly divided into fluorescence *in situ* hybridization and fluorescence *in situ* sequencing. Fluorescence *in situ* hybridization uses labeled fluorescent probes to determine RNA/DNA abundance in tissues or cells, and the first example of imaging a single RNA species can be attributed to single-molecule fluorescence *in situ* hybridization (smFISH) [14], which provides absolute quantification of the copy number and localization of RNA molecules in cells. However, due to the limitation of fluorescence signal overlap, it can only be used for imaging low-abundance RNA. Sequential fluorescence *in situ* hybridization (seqFISH) [15] is a multiplexed smFISH with tens of thousands of distinct transcripts detected. Through continuous rounds of hybridization, imaging, and probe stripping, a single transcript is detected multiple times, which improves the target detection throughput. The existing fluorescence *in situ* hybridization methods have advantages in spatial resolution and target detection throughput, but they are expensive and time-consuming due to the need to synthesize a large number of fluorescent probes and can only measure known target transcripts. 

In contrast, fluorescence *in situ* sequencing methods use single-base-specific fluorescence hybridization and DNA ligation reactions to determine the sequences of known or unknown transcripts in tissues. The representative technology is *in situ* sequencing (ISS) [3], which provides a highly multiplexed *in situ* detection scheme for different types of RNA by color sequence coding multiple imaging, which is not limited by the number of spectral and microscopic detection channels. Although the ISS method achieved *in situ* sequencing of the transcripts for the first time, only the known sequences of the target sites can be determined. Fluorescent *in situ* sequencing (FISSEQ) technology [16] uses random primers to generate complementary DNA (cDNA) and then circularizes the cDNA by single-stranded DNA cyclase. After magnifying the rolling circle amplification (RCA) signal, the transcript itself is sequenced to obtain the transcript sequence information, and the RNA expression at the transcriptome level can be located and analyzed simultaneously in a non-targeting manner. Image-based spatial transcriptomics technology theoretically has high spatial resolution, but due to factors such as optical crowding, there is only a low gene number detection throughput. 

With the rapid development of *in situ* capture technology, high-throughput single-cell multi-omics sequencing technology, and increasingly sophisticated microfluidic manipulation technology, spatial omics sequencing methods have dramatically developed. It has the advantages of high throughput, high sensitivity, a large tissue area for analysis, and the ability for multimodal analysis, making it an important technique for single-cell spatial resolution analysis. Therefore, this review mainly discusses the *in situ* capture spatial omics technology based on sequencing. Firstly, the spatial transcriptomics methods, including ST, Slide-seq, HDST, Stereo-seq, sci-Space, and Pixel-seq, are introduced in detail, focusing on the spatial barcode array strategy and the spatial information acquisition strategy applying microfluidic chips, and each method is compared from the perspectives of spatial resolution, simplicity of operation, and cost. Then we will focus on multi-omics spatial sequencing technology, including DBiT-seq, Spatial-CUT&Tag, Spatial-ATAC-seq, Spatial-CITE-seq, and Stereo-CITE-seq. Furthermore, two mature commercial spatial omics sequencing technologies and their applications are introduced. Finally, we point out the shortcomings of current spatial omics sequencing technologies and the direction of future technological progress.

## 3. Spatial Transcriptomics Sequencing Methods Based on Barcoded Array Chips

Spatial transcriptomics sequencing methods encode spatial location information into transcripts by *in situ* capture before sequencing and then retrieve transcripts and their spatial location information through high-throughput sequencing. Therefore, there are two key procedures to achieve spatial transcriptomics sequencing: preserving spatial information and reconstructing spatial information. To preserve spatial location information, the core step is to construct a barcode microarray chip with spatial location information. The following content will describe in detail the construction method of a barcode microarray loaded with spatial information and how to reconstruct spatial location information through sequencing.

### 3.1. ST Method

**Figure 1:**
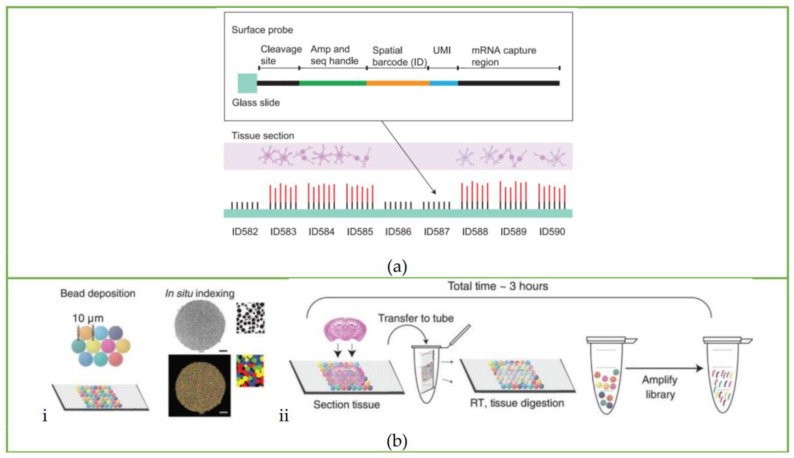

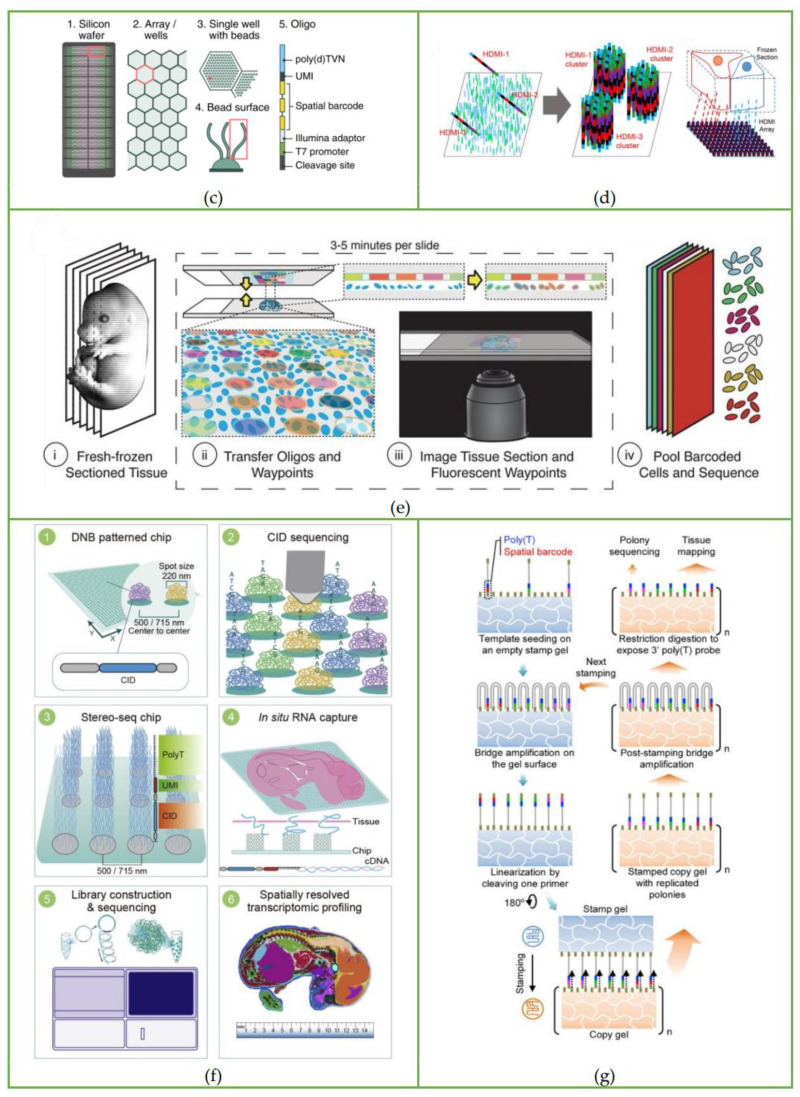
Workflow of spatial transcriptomics sequencing methods: Schematic of the workflow of the (**a**) ST method. Reprinted from ref. [17]. (**b**) Slide-seq: (**i**) schematic of array generation and (**ii**) schematic of sample preparation. Reprinted from ref. [18]. (**c**) HDST method: Barcoded bead array generation process. Reprinted from ref. [19]. (**d**) Seq-Scope: Different HDMI cluster formation processes and HDMI arrays capture RNA released from the overlying frozen section. Reprinted from ref. [20]. (**e**) sci-Space: Arrayed single-stranded oligos transferring to nuclei and imaging process. Reprinted from ref. [21]. (**f**) Stereo-seq: The whole pipeline of Stereo-seq, including DNB-patterned array chip design, spatial coordinates sequencing, capture probe preparation, *in situ* RNA capture, library construction and sequencing, and finally the data analysis process. Reprinted from ref. [22]. (**g**) Pixel-seq: Schematic of amplifiable DNA stamping. Reprinted from ref. [23].

In 2016, Joakin Lundeberg’s group first introduced a spatial sequencing method (hereinafter referred to as the ST method) [17] that used a barcode microarray to capture transcripts *in situ* and then retain the location information of transcripts, which opened the curtain of spatial transcriptomics sequencing. The ST method incorporates positional molecular barcodes during the process of cDNA synthesis. This process began with loading fixed tissue sections on the glass slide immobilized with reverse transcription primers containing spatial information, and the slides were stained and imaged for tissue morphology. The tissue section was then permeabilized, and *in situ* reverse transcription was performed. After tissue removal, spatially barcoded cDNAs were released from glass slides and prepared for next-generation sequencing (NGS) to obtain gene and spatial information. The array features of the ST method are shown in Figure 1a.

**Figure 2:**
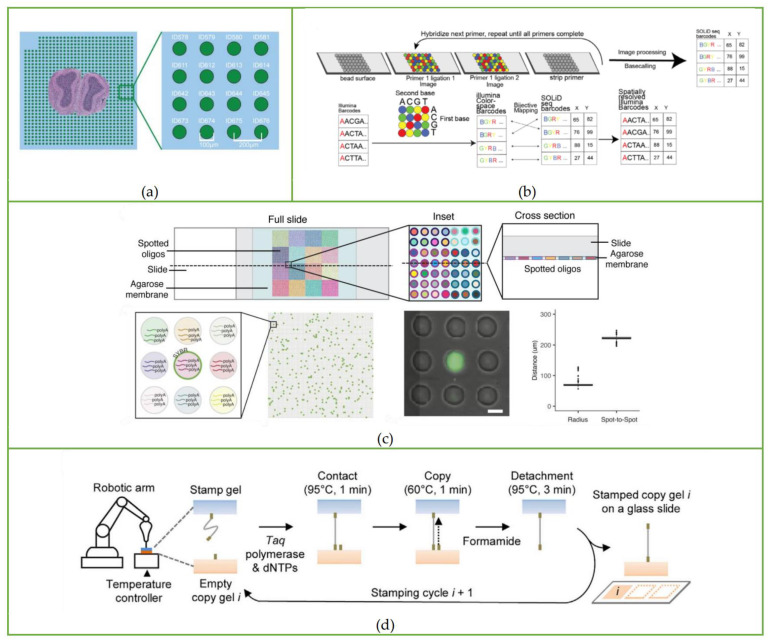
Microfluidic chips and autonomous devices to fabricate a barcoding array. (**a**) Spatial barcoding array of the ST method. Reprinted from ref. [17]. (**b**) Barcoded surfaces (“puck”) of Slide-seq. Reprinted from ref. [18]. (**c**) Space grids of sci-Space. Reprinted from ref. [21]. (**d**) Pixel-seq gel-to gel DNA copying progress is automated with a stamping device. Reprinted from ref. [23].

The barcoded array used in the ST method consists of barcoded capture primers printed on a 6.2 mm × 6.6 mm glass plate, as shown in Figure 2a. The diameter of each spot is 100 μm, and the distance between the center points of a single spot is 200 μm. The diameter of each spot is much larger than the diameter of a single cell, which means this method is far from the level of single-cell resolution. In addition, when making a glass slide coated with a barcoded array, the process is costly and complicated.

### 3.2. Slide-Seq 

Instead of the laborious method of printing barcoded capture primers on glass slides, Rodriques et al. [18,24] attached oligonucleotides with spatial barcodes to the beads in the solution and then randomly deposited them onto glass slides to form a single layer, thereby developing the Slide-seq method. This is the second spatial transcriptomics sequencing method published after the ST method. The barcoded beads of this method are synthesized by ChemGenes Corporation and dispensed onto the cover glass in solution. After drying, a single layer of ‘puck’ is formed on the cover glass covered with rubber, which is shown in Figure 1b(i) and Figure 2b. The fresh-frozen tissue sections are placed on the surface of the puck, and the beads can capture the mRNA released from the tissue. The sample preparation process is displayed in Figure 1b(ii). Slide-seq can achieve a spatial resolution of 10 μm, which is nearly 10 times higher than the initial ST method, and can detect the local gene expression in a single cell.

Although Slide-seq makes it much easier to establish an array with spatial barcode information, the process of decoding spatial information is complicated, as shown in Figure 2b. Slide-seq decodes the spatial barcode sequence by the SOLiD (sequencing by oligo nucleotide ligation and detection) technique, which repeats the primer hybridization, ligation, and stripping processes. After each ligation, fluorescent signals are recorded, and then the barcode information of each bead is obtained after basecalling. After obtaining SOLiD barcodes, a Matlab program is used to map them to Illumina barcodes for subsequent library analysis, and the main cost of Slide-seq is on the Illumina Novaseq platform (~$200–500/3 mm puck).

### 3.3. HDST Method

Then, the high-definition spatial transcriptomics (HDST) method developed by Vickovic et al. [19] further increased the spatial resolution to 2 μm. Compared with the Slide-seq method, which directly deposits beads on glass slides, the HDST method uses a magnetic bead array to deposit barcoded poly(d)T oligonucleotides into 2-micrometer wells randomly, which is shown in Figure 1c, and then the frozen tissue is placed on the barcoding board, where the mRNA is captured and profiled by RNA sequencing. However, the process of decoding spatial barcode information is still complicated. The sequential hybridization method is used: each round includes hybridizing a set of complementary oligonucleotides, recording the fluorescence on the entire slide, and stripping the oligonucleotides for the next round. These procedures are repeated until all spatial barcodes are decoded. Due to the use of red, green, and colorless (dark) fluorescence as labels, the hybridization process needs to be repeated log_3_N times. Taking the three spatial barcodes in the article as an example, it takes 14 cycles to fully decode the spatial barcodes on each bead, making it laborious and complicated.

### 3.4. Seq-Scope

Based on the Illumina sequencing chip, Cho et al. [20] further improved the resolution of spatial transcriptomics sequencing to the submicron level. Seq-Scope is a solid-phase amplification method based on the Illumina sequencing platform that is divided into two steps. The first step is to generate a spatial barcoded RNA capture molecule array and an association map of barcoded sequences and spatial array coordinates. The specific procedure is to use the Illumina sequencing platform to amplify the single-stranded synthetic oligonucleotide library, and the library is amplified on the lawn to form clusters. Each cluster has thousands of oligos that are consistent with the original seed oligos. Such clusters are called high-definition map coordinate identifier sequences (HDMI sequences), as shown in Figure 1d. Then the relationship between the HDMI sequence and the spatial coordinates of each cluster is determined by sequencing and synthesis using real-time analysis software. Finally, the oligo of each cluster is treated to expose the nucleic acid capture region, resulting in an HDMI array that can be used to capture mRNA. The second step of sequencing is to place the tissue on the HDMI array and capture the mRNA released from the tissue to generate cDNA. The second strand is synthesized according to the first strand using the adaptor-tagged random primer. Finally, the secondary strands are collected for library construction, PCR reactions, and sequencing. The tight arrangement of the RNA-capturing barcode clusters assures that the Seq-Scope has a resolution comparable to that of optical microscopy: ~0.5–0.8 μm. At the same time, it also has excellent transcriptome capture output: ~23–27 UMIs/μm^2^ and ~4700 UMIs/cell.

The disadvantage of Seq-Scope is also obvious: it can only capture transcriptomes with poly-A tails and consequently cannot detect whole transcriptomes. Moreover, the cost of this method is also high: the production cost of a MiSeq-based HDMI array reaches ~$150/mm^2^, and the time period from two-step sequencing to library preparation is three days. However, these two shortcomings can be improved by reducing the sequencing cost and accelerating the sequencing time.

### 3.5. Sci-Space

Sci-Space [21] is a spatial transcriptomics sequencing method developed by Jay Shendure’s group based on sci-RNA-seq [25], sci-RNA-seq3 [26], and sci-Plex [27]. The sci-Plex was developed based on the phenomenon that single-stranded DNA could diffuse into cell nuclei, serving as a hash oligo to label single nuclei. According to this principle, sci-Space uses a microarray scanner to produce space grids with hash oligos and fluorescently labeled reference points on an agarose film, as shown in Figure 2c. When the tissue section is attached to the space grid, hash oligos diffuse into the cell nuclei. During reverse transcription, poly(A)-tailed hash oligos and transcripts are captured by primers with the same nuclei-specific indices, so that the transcripts and hash oligos from the same nuclei can be identified according to nuclei-specific indices during sequencing. The spatial location of the cell can be further determined by oligo-hashing. The whole workflow of the sci-Space method is shown in Figure 1e.

The sci-Space method is different from the previous strategies of using poly(d)T primers to capture mRNA: it uses short single-stranded DNA to label single nuclei, providing a new solution to add spatial barcode sequences. The spatial resolution of this method is about 200 μm, leading to the consequence that the interaction between adjacent cells cannot be detected. However, since transcripts are fixed in nuclei, which remain intact during barcoding, this method still has single-cell resolution.

### 3.6. Stereo-Seq

The Stereo-seq technology [22] developed by BGI Genomics combines self-assembled DNA nanoballs (DNBs) to prepare spatial coding arrays to further improve spatial resolution. The DNB template includes random barcodes and adaptors, and the spatial coordinates of each sphere can be obtained by *in situ* sequencing. To produce Stereo-seq chips, DNBs are generated by rolling circle amplification and loaded onto the patterned chips shown in Figure 1f. The diameter of the spots on the DNB chip is about 220 nm, and the distance between the spots is 500 or 715 nm. There are 400 spots per 100 μm^2^, reaching 4^25^ different spots. Then, UMI and poly(d)T are hybridized and ligated to each spot, and the tissue is placed on the chip for *in situ* RNA capture.

The spatial transcriptomic information is obtained by sequencing. The whole pipeline of Stereo-seq is shown in Figure 1f. The spatial resolution of Stereo-seq can reach 220 nm, and the number of captured UMI is also improved compared with Seq-Scope: the diameter of Stereo-seq is 1450/10 μm, while Seq-Scope is 848/10 μm. In terms of cost, the spatial capture array is significantly lower than the Seq-Scope: The MiSeq-based HDMI array of Seq-Scope requires ~$150/mm^2^, while the DNB capture chip of Stereo-seq only requires ~$35/mm^2^, but the sequencing cost of this method still accounts for the main part: ~$800. Meanwhile, this method has a large overall capture area of about a centimeter, facilitating large-field spatial transcriptomic analysis.

### 3.7. Pixel-Seq

The above methods can be classified as sequencing-dependent arrays. That is, each barcode array needs to be sequenced once to obtain spatial information, which is time-consuming and laborious. Fu et al. [23] recently developed the pixel-seq (poly-indexed library-sequencing) method, which uses an enzymatic method to copy the barcode-patterned gels as an elastic seal to replicate many known barcode arrays. It greatly reduces costs and saves time. To fabricate the stamp gel, the oligonucleotides with spatial barcodes are first seeded on the blank stamp gel and amplified into a cluster by bridge PCR. The reverse strand and the link parts of the primers are cut, thus leaving only the forward strand covalently linked to the chip. Then the stamp gel is inverted 180 degrees to obtain a copy gel by DNA polymerase catalytic chain extension, and subsequently the copy gel is amplified by bridge PCR. The 3’poly(d)T primer is exposed by TaqI digestion and placed on the tissue for spatial barcoding. The DNA stamping chemistry process is shown in Figure 1g. Some of these copy gels are used as stamps for the sequencing, some are used for polony sequencing to create a spatial barcode map, and most are used to attach tissues.

It is worth noticing that the autonomous device built for the stamping process includes a desktop robotic arm to adjust the seal position, a thermal cycler to control the gel temperature, a digital balance to monitor the stamping pressure, and a fluid system to amplify DNA, which is shown in Figure 2d. Therefore, this method made great progress in spatial transcriptomics techniques in terms of automation, cost savings, and efficiency.

The advantages of this method are obvious: since both primers and templates are covalently attached to the gel, diffusion is prevented. Enzymatic replication does not consume the template, and 50 cycles of replication only lead to 15% feature loss, showing that repeated stamping is robust. Moreover, each copy product is the same, so the copy gels obtained from the same batch of stamping only need to be sequenced once. Due to the reduction in spatial barcode sequencing, the time and economic costs are also greatly reduced. For example, the fabrication cost of Stereo-seq requires ~$35 per square millimeter, while stamp gel only requires ~$0.06 mm^2^; for array fabrication time, Seq-scope and Stereo-seq take 17 and 9 h, respectively, while pixel-seq only takes 7 h.

## 4. Spatial Multi-Omic Sequencing Methods Based on Microfluidic Chips

Single-cell multi-omics technology is at the forefront of the development of single-cell analysis technology, and it is also the inevitable trend of the development of single-cell omics [28,29]. It is also rated by Nature Methods as the technical progress of 2019 [30]. The above methods are limited to spatial transcriptomics, and different epigenetic markers, such as DNA methylation [31] and protein modification, also affect the way cells read the genome, thus affecting the transcription of the genome. Based on the characteristics of chromatin accessibility, the Assay for Transposase-Accessible Chromatin Sequencing (ATAC-seq) technology [32] inserts the Tn5 transposase carrying sequencing information into chromatin. The transposase can insert into the open region of chromatin and mark the DNA sequence. After sequencing, gene regulation can be explained from the perspective of epigenetics. In addition, whole genome sequencing [33,34,35,36] and proteomic sequencing [37,38] are also used in combination with single-cell transcriptomics sequencing. Single-cell multi-omics technology breaks through the monotony of previous single-cell transcriptomics sequencing by analyzing cell characteristics more comprehensively and having great advantages in discovering and identifying new cell subtypes. Therefore, single-cell multi-omics technology is also the development direction for spatial omics sequencing. At present, some studies have explored multi-omics sequencing at spatial resolution.

### 4.1. DBiT-Seq

The deterministic barcoding in tissue for spatial omics sequencing (DBiT-seq) technology developed by Fan Rong’s group [39] pioneered the study of spatial multi-omics sequencing. Different from the previous usage of spatial microarrays or nanoballs to capture and release mRNA, DBiT-seq uses microfluidic technology to perform *in situ* spatial barcoding of cells to achieve joint measurement of the spatial transcriptome and protein. 

**Figure 3:**
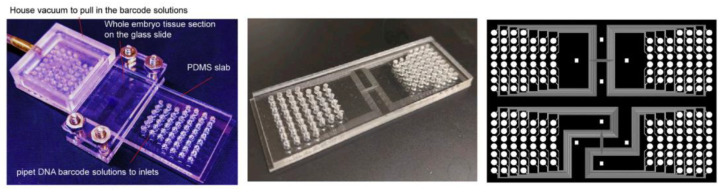
The microfluidic device used in DBiT-seq, Spatial-CUT&Tag, and spatial-ATAC-seq. Reprinted from ref. [39].

The utilization of microfluidic PDMS chips shown in Figure 3 greatly simplifies the barcoding array fabrication process. The common shortcoming of the above-mentioned spatial transcriptomics sequencing method is that the barcoding process is very sophisticated, and both capture tissue mRNA on a solid-phase substrate, while DBiT-seq proposes a totally different idea: direct spatial barcoding of biomolecules on tissues. To be specific, a microfluidic PDMS chip containing 50 parallel microchannels is placed on a glass slide with tissue on it to introduce oligo-dT-tagged DNA barcodes A1–A50; after *in situ* reverse transcription, the microfluidic chip perpendicular to the first chip channel is placed on the tissue slide, and the second DNA barcode B1–B50 is introduced, which is ligated at the intersection of barcode A to form a distinct combination array as AiBj (i = 1~50, j = 1~50), totally generating 2500 spatial barcode pixels. 

**Figure 4:**
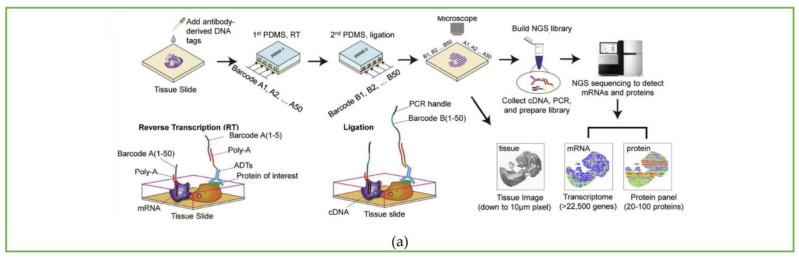

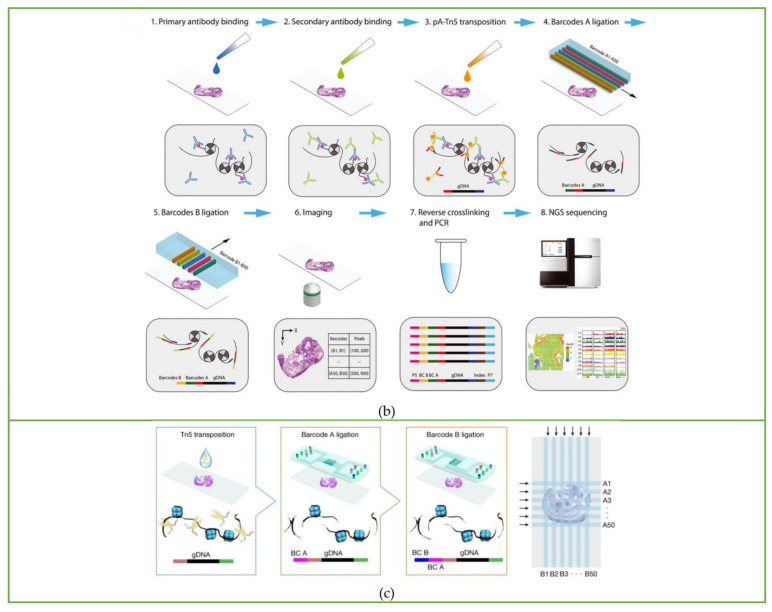
Workflow of spatial multi-omics sequencing methods. Schematic of the workflow of (**a**) DBiT-seq, including the barcoding process and strategy of binding with mRNA or protein, along with library construction and the NGS sequencing process. Reprinted from ref. [39]. (**b**) Spatial-CUT&Tag steps: primary antibody binding, secondary antibody binding, pA-Tn5 transposition, barcode A and B ligation, imaging, reverse crosslinking, PCR, and finally NGS sequencing. Reprinted from ref. [40]. (**c**) Spatial-ATAC-seq. Tn5 transposition is performed in tissue sections, followed by *in situ* ligation of two sets of DNA barcodes. Reprinted from ref. [41].

Afterwards, the cDNA is extracted from the digested tissue, and then, after PCR amplification and library preparation, NGS can be performed. The spatial expression map will be reconstructed by identifying the spatial barcode AiBj connected to the cDNA. In addition to transcriptomics sequencing, they used antibody-derived DNA tags (ADTs) to perform spatial localization and quantitative analysis of different proteins before microfluidic barcoding, resulting in the construction of a multi-omics gene expression spatial map. The whole process is shown in Figure 4a.

DBiT-seq has clear superiority in terms of data quality compared to Slide-seq with the same size pixel (10 μm), which can only detect ~150 genes; DBiT-seq can detect an average of 2068 genes per pixel. Previous methods using spatial microarrays or nanoballs required a long and complex bead decoding process, such as sequential hybridization or SOLiD sequencing, while DBiT-seq omits these steps and greatly reduces time and cost. The chip is directly clamped onto the tissue, and the solution is directly introduced into the chip without additional microfluidic control, which is easy to operate. At the same time, this method has good compatibility: it does not need to dissociate tissue to obtain mRNA and can also be achieved with existing formalin-fixed tissue sections, which greatly expands the application range of the technology.

This method also has drawbacks: even if the DBiT-seq resolution is close to the single-cell level, it cannot directly detect a single cell. In terms of chip design, since the thickness of the tissue is greater than the thickness of the microchannel of the chip, it is possible to cause channel blockage, and the flux of the channel is small, resulting in a limited number of barcodes. The above channel problems can be compensated for by improving the design of the microfluidic chip.

### 4.2. Spatial-CUT&Tag

Based on DBiT-seq, Fan Rong’s group continued to extend this method to study histone modification at spatial resolution [40]. The Spatial-CUT&Tag method combines in-tissue deterministic barcoding technology with cleavage under targets and tagmentation (CUT&Tag) chemistry. The first antibody is added to the fixed tissue section to bind to the target histone modification, and then the second antibody is combined with the first antibody to enhance the tethering effect of pA-Tn5 transposase. After the transposase is activated, the ligation linker is inserted into the genomic DNA (gDNA) at the histone marker antibody recognition site. Using the same method as DBiT-seq, barcode A and barcode B are passed into the microfluidic chip to hybridize with the adapters *in situ*. Then, the tissue is imaged to construct a map of histomorphology and the spatial transcriptome. Finally, the DNA fragments are released by reverse cross-linking and collected to complete library construction. The schematic workflow is displayed in Figure 4b.

The resolution of Spatial-CUT&Tag is 20 microns, which elevates the transcriptome and proteome of a single cell to the spatial level and analyzes the epigenetic mechanism of tissue development without bias, which is of great significance for studying the mechanisms of development and disease.

### 4.3. Spatial-ATAC-Seq Strategy

The spatial-ATAC-seq method developed by Deng et al. [41], also from Fan Rong’s group, for the first time achieved the study of chromatin accessibility *in situ*, opening a new perspective for the emerging field of spatial omics. Spatial-ATAC-seq uses microfluidic chips to perform spatial two-dimensional barcoding of tissues and combines it with ATAC-seq technology to achieve genome-wide chromatin accessibility analysis. The specific process is as follows: first, Tn5 transposase and adapters containing ligation linkers are inserted into accessible genomic loci, and then, as in the DBiT-seq method, barcodes A1–A50 and B1–B50 are introduced into the microfluidic chip, respectively, which are connected to the end of the Tn5 oligo 5′ end as shown in Figure 4c. Then the tissue sections are imaged to correlate spatial chromatin with histomorphology. After the tissue is ligated with the barcodes, the barcoded DNA fragment is released by reverse cross-linking, then amplified by PCR, and the library is prepared for sequencing.

Compared with spatial-CUT&Tag studies on histone modification, spatial-ATAC-seq provides genome-wide chromatin accessibility. In terms of resolution, spatial-ATAC-seq basically reached the single cell level: 20 μm pixel size. Similar to DBiT-seq, it does not need to dissociate the tissue, and the pixel containing only one nucleus can be selected for subsequent analysis by immunofluorescence imaging.

### 4.4. Spatial-CITE-Seq

Recently, Fan Rong’s group further developed spatial transcriptomics integrated with proteomics technology called Spatial-CITE-seq [42]. Compared with DBiT-seq, which can detect 22 proteins at a time, Spatial-CITE-seq enlarges that number to ~200–300 so that it realizes high-plex protein and transcriptome co-mapping. The workflow of Spatial-CITE-seq is almost the same as that of DBiT-seq: ~200–300 ADTs are applied to the tissue section, followed by in-tissue synthesis of cDNAs of endogenous mRNAs and introduced ADTs using parallel channels A1–A50. Then barcodes B1–B50 are ligated with barcodes A1–A50 by placing perpendicular microchannels. Finally, barcoded DNA is collected, purified, amplified, and prepared for paired-end NGS sequencing. The microfluidic device used to add barcodes is the same as the DBiT-seq shown in Figure 3.

Spatial-CITE-seq is the highest multiplexing for spatial protein profiling among both imaging-based protein mapping and sequencing-based methods, and it can expand to >1000-plex protein detection. However, the resolution cannot reach the subcellular level as with imaging-based methods, which is still one of the main advantages of imaging-based methods.

### 4.5. Stereo-CITE-Seq

BGI Genomics also developed a transcriptomics and proteomics combined sequencing technology called Stereo-CITE-seq [43] based on their original spatial transcriptome method, Stereo-seq. Utilizing the high resolution (500 nm) characteristics of Stereo-seq, Stereo-CITE-seq achieves high spatial resolution multi-omics profiling. Stereo-CITE-seq applies similar strategies to CITE-seq, using poly(dT) oligos to capture ADT and mRNA. Then, ADT and mRNA are reverse-transcribed. After release, ADT and cDNA are amplified and constructed separately.

Compared to Spatial-CITE-seq, which can detect hundreds of ADTs at one time, Stereo-CITE-seq can only detect 17 plex ADTs. However, the 500-nanometer ultra-high resolution is the highlight of this method. The shortage of Spatial-CITE-seq and Stereo-CITE-seq is that they can only detect surface proteins. The detection of intracellular and extracellular proteins should be considered in the future.

The characteristics of the *in situ* capture methods mentioned above are summarized in detail in Table 1.

## 5. Commercialization of Spatial Sequencing Methods

**Figure 5:**
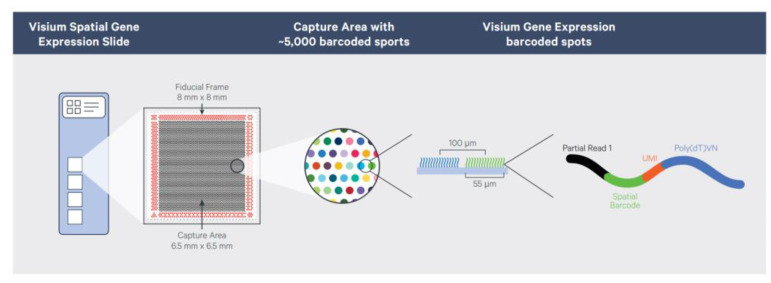
Composition of the Visium spatial gene expression slide. Reprinted from ref. [54].

The existing mature commercial transcriptomics technologies mainly include Visium from 10x Genomics, GeoMx from NanoString, and Stereo-seq, which was launched by BGI Genomics and has the highest resolution. Visium chip technology is a commercial method introduced in 2018 by 10x Genomics, which acquired it from the ST method. It combines spatial transcriptomics technology with the H&E staining method to provide cell morphological information and the immunofluorescence staining method to provide gene and protein expression patterns of the same tissue section. The composition of the Visium slide is displayed in Figure 5. Compared with the ST method, Visium has improved the resolution: the area where tissues are placed is 6.5 × 6.5 mm^2^, with 5000 sites distributed. Each site is 55 μm in width, and the sites are separated by 100 μm. The entire process, from sample preparation to library construction for sequencing, can be completed in one day. Applying the Visium platform, many articles about cell subtype identification [44,45], tumor heterogeneity [46,47,48], cancer metastasis mechanisms [49], cell development mechanisms [50], biomarker discovery [51,52], and immunology [53] were published.

The GeoMx Digital Spatial Profiler [55] of Nanostring Biotechnology is a new generation of spatial multi-target analysis systems. It is the only platform on the market with high throughput, multi-omics, and an elastic selection of regions of interest. It combines fluorescence imaging, immunohistochemistry, and gene transcriptomics techniques to depict the most comprehensive and clear biological images for researchers. It solves the problem that traditional analysis platforms cannot simultaneously obtain morphological information and multiple target expression data sets. GeoMx Digital Spatial Profiler uses an exclusive patented technology to couple DNA oligos to antibodies or RNA. Each DNA oligo corresponds to a target, up to 96 protein targets or more than 1000 RNA targets. When the antibody or RNA binds to the target on the tissue, GeoMx Digital Spatial Profiler uses a laser to cut off the linker between the DNA oligo and antibody or RNA, thereby releasing the DNA oligo for further quantification.

Compared with Visium, GeoMx DSP is a more microscopic and detailed spatial targeting research technology. DSP is based on multiple fluorescence results to target gene expression in target microregions. At the same time, the DSP million-level micromirror array can further subdivide the target into microregions. For example, the tumor immune microenvironment is divided into tumor areas and immune areas to accurately analyze the tumor microenvironment. Moreover, the DSP spatial transcriptome provides an implementation method for regional targeting research that effectively avoids the pressure of data mining caused by the undifferentiated detection of whole slices and therefore greatly improves the detection efficiency. Using this method, hundreds of articles have been published. For example, the use of GeoMx Digital Spatial Profiler led by David Rimms [56] to discover novel predictive markers for melanoma immunotherapy patients occupied the cover of Clinical Cancer Research in October 2019. This technology has good application performance in spatial heterogeneity analysis [57], tumor microenvironment research [58], Immune system response research [59,60], neuroscience research [61], and disease pathological process research [62,63]. However, the resolution of GeoMx DSP is hundreds of microns, and it still does not reach single-cell resolution [64].

The comparison of 10x Genomics Visium and NanoString GeoMx DSP technology is shown in Table 2.

## 6. Discussion and Future Perspectives

### 6.1. Limitations and Possible Improvements of Barcoded Array Chips

Different methods adopt different strategies when making microfluidic barcoded array chips. The design and fabrication of microfluidic array chips will directly affect the simplicity and cost of the methods. The existing strategies for making barcoded arrays have several defects: First, the fabrication process is complicated and laborious. The ST method is to covalently attach the barcoded reverse transcription oligonucleotide primers on the glass slide as capture probes. The Slide-seq method is to randomly deposit the beads attached to the spatial barcodes on the glass slide. The HDST method is to deposit the magnetic beads with primers into microwells. The Stereo-seq method is to make self-assembled DNA nanospheres with random barcodes and adapters. It can be seen that whether it is to make primers and probes and attach them to the expected spot or to make barcoded microspheres, the procedure is technically demanding and time-consuming. Secondly, most barcoded array chips need to be decoded in order to obtain spatial information because random primers are used when fabricating spatial arrays or chips. Coupled with the last step of sequencing, the whole process requires two sequencing steps. For example, Slide-seq uses SOLiD sequencing, which itself requires multiple cycles of primer hybridizations, and the operation is laborious. Another case is the HDST method, which requires continuous hybridization, stripping, and imaging to decode spatial barcodes. Including subsequent Seq-Scope and Stereo-seq technologies, high-throughput sequencing machines are required to perform de novo sequencing to obtain spatial information, which is the most cumbersome step of different technologies. Thirdly, there is a conflict between high resolution and making a barcoded array with a known sequence. To be specific, existing high-spatial-resolution methods such as Seq-Scope and Stereo-seq, which have single-cell or sub-cell resolution, need two sequencing steps. While the deterministic barcode methods using microfluidic chips, which need only one sequencing, are difficult to achieve single-cell resolution. Fourthly, due to the limitations of chip size and the number of barcodes, the area of capture is limited, which hinders large-scale spatial omics sequencing. 

It can be seen from the above description that much attention should be paid to optimizing the design and fabrication of the barcoded array. Here, we propose the following ideas that can be considered: Sequencing costs account for the majority of the total cost, so the determined and known barcoded array is the trend of future spatial omics sequencing technology because it omits the long and complex decoding process. An example is DBiT-seq technology, in which fixed spatial barcodes are sequentially introduced on the chip and the barcode information of each spot is determined and known. Then, to increase the area of detectable tissues, current microfluidic control technology can be utilized to better arrange the barcode sequence to increase the number of spatial spots. Another is developing a stable barcode hybridization technology to ligate barcode fragments multiple times to increase the diversity of barcodes. Moreover, to simplify barcode sequence adsorption and desorption procedures and improve efficiency at the same time, one of the considerations is to convert chemical attachment on chips to physical adsorption. In detail, DNA immobilization techniques contain physical adsorption methods such as hydrophobic interaction, electrostatic interaction, pH value change, and so on. This way, we can not only omit the complex chemical attachment process when making barcoded array chips but also facilitate the release and transfer of the spatial barcodes to cells conveniently.

### 6.2. Bridging the Gap between Single-Cell Sequencing and Spatial Omics Sequencing

Although spatial omics sequencing relies on the development of single-cell sequencing methods, there is still a gap between existing spatial sequencing methods and traditional single-cell sequencing in terms of gene detection efficiency and accuracy. This is because traditional single-cell sequencing can capture transcripts of different genes almost without deviation, which makes it easier to achieve separation between single cells. In contrast, spatial omics sequencing has problems such as different resolutions of capture points and potential transcript diffusion, which make it difficult to distinguish single cells. Therefore, it is an essential development direction to apply the existing advanced high-throughput single-cell sequencing methods to spatial sequencing technology.

### 6.3. Multi-Omic Sequencing Apply in Spatial Omics Sequencing

Traditional single-cell technology has developed rapidly towards multi-omics. Joint multi-omics sequencing overcomes the limitations of a single modality, providing more comprehensive information on gene regulation. By now, a number of multi-omics sequencing technologies have emerged: transcriptome integration with the genome [65]; transcriptome integration with chromosome accessibility [66]; transcriptome integration with histone modifications [67]; and transcriptome integration with the methylome [68]. In addition to the integration of two omics, the integration of three or more modalities has become a trend. scNOMeRe-seq, developed by Wang et al. [69], can profile genome-wide chromatin accessibility, DNA methylation, and RNA expression in the same individual cell, comprehensively analyzing the regulatory relationships of mammalian preimplantation embryos at multiple levels in a single cell.

The existing spatial multi-omics sequencing methods are limited. It is not satisfactory, no matter the coverage of omics or the quality of multi-omics sequencing. In theory, multi-omics sequencing from ordinary single-cell sequencing can be applied to spatial omics. However, given the features of spatial omics sequencing, spatial multi-omics sequencing will be more tricky than ordinary multi-omics sequencing. This is because once the spatial location of the cell is destroyed, the tissue cannot be used again. Therefore, it is necessary to obtain other omics information before tissue dissociation. Integrating multiple omics without destroying the spatial form of the organization is the key to applying multi-omics sequencing to spatial profiling.

### 6.4. The Needs of Real Three-Dimensional Spatial Profiling

Another problem with current spatial sequencing technology is that it still uses two-dimensional profiling, which is not real three-dimensional profiling. There is a research group [70] using continuous tissue sections for two-dimensional spatial gene expression analysis and then reconstructing the obtained two-dimensional gene expression map to acquire a three-dimensional gene expression map. However, there are still problems, such as the inability to guarantee the quality of sections and the difficulty in obtaining a perfect three-dimensional structure. To solve this problem and realize true three-dimensional *in situ* capture, the idea of *in situ* capture should be converted from placing tissue slices on barcoded array chips to penetrating barcodes into tissues. 

### 6.5. Limitations in the Application of Spatial Omics Sequencing and Possible Solutions

The application of spatial profiling technology is of particular concern. In theory, any tissue containing active mRNA or intact, well-fixed mRNA is suitable for spatial sequencing technology. However, due to the defects of existing methods, the application of spatial omics is limited. Take the example in our experimental process: when choosing the tissues, try not to choose the degraded FFPE tissues or the fragile fresh frozen tissues. When placed on a slide or chip for analysis, the tissue section is easy to tear and deform. Therefore, rare and valuable tissue samples or tissues that need serial section sequencing may have technical operation problems. At the same time, low-resolution spatial barcoded chips will cause important information to be missing, especially when applying some porous tissue sections. 

It can be seen that to expand the spatial sequencing application range, resolution and gene detection number are key points. To improve this, two directions can be taken: spatial barcoded array design and last-step single-cell sequencing. Barcoded oligo sequences in liquid have diffusion issues, which will hinder the tight arrangement of the barcoded array, thereby further reducing resolution. The use of solid-phase microspheres or microarray sample applicators may alleviate this problem. The high-sensitivity sequencing method is also another focus to improve the quality of spatial sequencing technology. By simultaneously improving the fabrication of the upstream spatial array and the downstream sequencing sensitivity, the application range will become wider and wider.

### 6.6. The Need for More Advanced Commercial Spatial Profiling Technology

Lastly, spatial omics technology is widely desired; however, many researchers have a relatively high threshold to operate and obtain spatial sequencing information because of the complexity of the operation, leading to a huge demand for commercial spatial omics sequencing platforms. At present, mature commercial platforms are developed using early spatial sequencing technology with limited performance. Therefore, the commercialization of high-resolution advanced spatial sequencing technology is also one of the development directions for spatial sequencing technology.

## 7. Conclusions

This paper reviews the frontier progress of spatial omics sequencing technology, summarizes it, and discusses the technical key points of each method, mainly focusing on the fabrication strategies of barcoded array chips utilizing microfluidic technology. After less than 5 years of development, spatial sequencing technology has made great progress, from the resolution of a few hundred microns to the nanometer level, from multiple hybridization sequencing to obtain spatial location information in one sequencing, from single transcriptomics sequencing to spatial joint omics sequencing. However, there are still shortcomings, such as low resolution and sensitivity, limited tissue coverage, and imperfect coverage of omics. In the future, with the development of the latest sequencing technology and more advanced microfluidic chips, it is expected to develop low-cost, high-throughput, high-resolution, and high-sensitivity single-cell spatial omics analysis methods that can obtain a more accurate description of individual cells and construct spatial maps more economically.

## Figures and Tables

**Table 1:** A summary of the various *in situ* capture spatial sequencing methods mentioned in this review.

Name	Publication Year	Omics Involved	Resolution	Single-Cell Level	Detection Area	Cost	Reference
ST method	2016	Transcriptomics	Spot diameter: 100 μm Center-to-center: 200 μm	Not single-cell level	6.2 mm × 6.6 mm	Not mentioned	Ståhl P.L., et al. *Nat. Methods*, 2016 [17]
Slide-seq	2019	Transcriptomics	Bead diameter: 10 μm	Nearly single-cell level	3 mm diameter puck	~$0.10 chip fabrication and ~$200–500 sequencing/3 mm puck	Rodriques S.G., et al. *Science*, 2019 [18]
HDST method	2019	Transcriptomics	Bead diameter: 2 μm Well diameter: 2.05 μm	Nearly single-cell level	5.7 mm × 2.4 mm	Not mentioned	Vickovic S., et al. *Nat. Methods*, 2019 [19]
Seq-Scope	2021	Transcriptomics	Center-to-center: ~0.6 μm	Single-cell/subcellular level	10 mm × 10 mm	Total ~$150/mm^2^	Cho C.S., et al. *Cell*, 2021 [20]
Sci-Space	2021	Transcriptomics	Spot radius: ~73 μm Center-to-center: ~200 μm	Single-cell	18 mm × 18 mm	Not mentioned	Srivatsan S.R., et al. *Science*, 2021 [21]
Stereo-seq	2022	Transcriptomics	Spot diameter: 0.22 μm Center-to-center: 0.5/0.715 μm	Single-cell/subcellular level	13.2 m × 13.2 cm	~$35 chip fabrication and ~$8 sequencing/mm^2^	Chen A., et al. *Cell*, 2022 [22]
Pixel-seq	2022	Transcriptomics	Polony diameter: 1 μm	Single-cell level	7 mm × 7 mm	~$0.06 chip fabrication and ~$60 sequencing/mm^2^	Fu X., et al. *Cell*, 2022 [23]
DBiT-seq	2020	Transcriptomics and proteomics	Microchannel width: 10/25/50 μm	Nearly single-cell level	5 mm × 5 mm for maximum	Not mentioned	Liu Y., et al. *Cell*, 2020 [39]
Spatial-CUT&Tag	2022	Transcriptomics and histone modification	Microchannel width: 20/50 μm	Not single-cell level	5 mm × 5 mm for maximum	Not mentioned	Deng Y., et al. *Science*, 2022 [40]
Spatial-ATAC-seq	2022	Transcriptomics and chromatin accessibility	Microchannel width: 20 μm	Not single-cell level	2 mm × 2 mm	Not mentioned	Deng Y., et al. Nature, 2022 [41]
Spatial-CITE-seq	2023	Transcriptomics and proteomics	Microchannel width: 25 μm	Single-cell level	2.5 mm × 2.5 mm	Not mentioned	Liu Y., et al. *Nat. Biotechnol*, 2023 [42]
Stereo-CITE-seq	2023	Transcriptomics and proteomics	Spot diameter: 0.22 μm Center-to-center: 0.5/0.715 μm	Single-cell level	13.2 cm × 13.2 cm	Not mentioned	Liao S, et al. *bioRxiv,* 2023 [43]

**Table 2:** Comparison of 10x Genomics Visium and NanoString GeoMx DSP.

	10x Genomics Visium	NanoString GeoMx DSP
Tissue compatibility	Embedding frozen samples or FFPE paraffin samples	Any sample, FFPE, or fresh frozen samples
Tissue area	6.5 mm × 6.5 mm	14.6 mm × 36.2 mm
Detection area	Capture area	Regions of interest (ROIs)
Resolution	1–10 cells/spot	>100 cells/ROI
RNA capture method	polyA capture or specific probe hybridization	Specific probe hybridization
Species limitation	Eukaryote	Human and mouse

## Data Availability

Not applicable.
